# Fluorescent Carbon Dots for Super-Resolution Microscopy

**DOI:** 10.3390/ma16030890

**Published:** 2023-01-17

**Authors:** Xiangcheng Sun, Nazanin Mosleh

**Affiliations:** Department of Chemical Engineering, Rochester Institute of Technology, Rochester, NY 14623, USA

**Keywords:** carbon dots, properties, super-resolution microscopy, probes and applications, conclusions and outlook

## Abstract

Conventional fluorescence microscopy is limited by the optical diffraction of light, which results in a spatial resolution of about half of the light’s wavelength, approximately to 250–300 nm. The spatial resolution restricts the utilization of microscopes for studying subcellular structures. In order to improve the resolution and to shatter the diffraction limit, two general approaches were developed: a spatially patterned excitation method and a single-molecule localization strategy. The success of super-resolution imaging relies on bright and easily accessible fluorescent probes with special properties. Carbon dots, due to their unique properties, have been used for super-resolution imaging. Considering the importance and fast development of this field, this work focuses on the recent progress and applications of fluorescent carbon dots as probes for super-resolution imaging. The properties of carbon dots for super-resolution microscopy (SRM) are analyzed and discussed. The conclusions and outlook on this topic are also presented.

## 1. Introduction

Optical microscopy, as a powerful tool, has been widely used in a variety of areas such as chemistry, biology, medical, energy, and materials sciences. Using fluorescence microscopy, fluorescence signals are collected by an objective lens and corresponding filters upon excitation of fluorophores and form resulting images. Fluorescence imaging has become an indispensable tool, especially for studying biological systems, due to the biocompatibilities, decent spatial and temporal resolution, high specificity, and imaging contrast [1,2,3]. Although powerful, the conventional fluorescence microscope is limited by the optical diffraction of light [4]. The optical resolution is approximately calculated as

(1) $$\Delta =\frac{\lambda }{2\mathrm{nsin}\theta \;}=\frac{\lambda }{2N.A.\;}$$

with λ the wavelength of emitted light, n the refractive index of the medium containing the sample, θ the angular aperture of the microscope, and N.A. the numerical aperture of the lenses. Therefore, conventional fluorescence microscopy has a lateral resolution limit of ~250–300 nm. In order to extend the resolution to smaller values, several techniques were developed, such as near-field microscopy, confocal and multiphoton microscopy, and structured-illumination microscopy. The best achievable resolution using these methods is about half of the diffraction-limited resolution, approximately 100 nm [4]. 

To go far beyond the diffraction limit and to further improve the optical resolution, two major approaches have been developed using spatially patterned excitation or single-molecule localization microscopy [4]. For the spatially patterned excitation approach, super-resolution is realized through introducing sub-diffraction-limit features in the excitation pattern. There are stimulated emission depletion (STED) microscopy, reversible saturated optically linear fluorescence transition (RESOLFT), and saturated structured illumination microscopy (SSIM), etc., in this approach [5]. The spatial resolution of these super-resolution techniques could be obtained with an order of magnitude improvement over the conventional optical microscopy. The other approach, the single-molecule localization-based super-resolution approach, takes advantage of photoswitching/activation and imaging a single-molecule to circumvent the problem of light diffraction; some examples of this approach are stochastic optical reconstruction microscopy (STORM), photoactivated localization microscopy (PALM), and fluorescence photoactivated localization microscopy (FPALM) [4,5,6,7]. Because of the development and importance of super-resolution (SR) imaging, the Nobel Prize in Chemistry in 2014 was awarded to Eric Betzig, William E. Moerner, and Stefan W. Hell. 

The advancement and success of super-resolution imaging have always relied on the discovery and application of bright and easily available fluorescent probes [5,6]. Native biological structures (although with intrinsic fluorophores sometimes) generally are not good enough for super-resolution imaging due to low contrast, poor photostability, limited emission, or lack of special properties (such as photoswitching or blinking). Therefore, extrinsic probes are necessary. Theoretically, almost any biomolecules of interest could be investigated with extrinsic fluorescent probes if attached specifically. Exploring for an appropriate extrinsic probe is thus ongoing. 

A series of fluorescent probes, such as fluorescent proteins, organic dyes, and quantum dots (QDs), have been extensively utilized for super-resolution imaging [5,7,8]. None of these materials are ideal or without imperfections. Fluorescent proteins are biocompatible with low cytotoxicity but exhibit low photon output and low photoswitching, which may result in low localization precision. The easily accessible organic dyes with tuned emission colors may be considered as good candidates for SRM; however, their applicability in SRM is restricted by rapid photobleaching and buffer dependency of blinking. When it comes to the QDs, their toxicity, high duty cycle, and rapid blinking restrict their applications, although they show good photophysical properties such as excellent quantum yield, high absorption coefficient, and brightness and also resistance to photobleaching. 

As one kind of novel fluorescent materials, carbon dots (CDs) were accidently discovered in 2004 by Srivens and co-workers after separation and purification of single-walled carbon nanotubes through arc discharge methods [9]. Since then, CDs have been prepared by a series of methods which are generally classified as bottom-up and top-down approaches [10,11]. In bottom-up approaches, CDs are prepared by assembling molecular precursors under various reaction conditions, such as solvothermal, hydrothermal, and microwave-assisted conditions. For top-down approaches, CDs are obtained by breaking down larger pieces of material into the desired nanostructures under conditions of electrochemical, laser ablation, arc discharge, etc. CDs generally include carbon nanodots, graphene quantum dots (GQDs), and carbonized polymer dots [11]. CDs have already found abundant applications in chemosensing, biosensing, imaging, light-emitting devices, catalysis, and the medical field, owing to properties such as biocompatibility, facile synthesis, low cost, tuned emission properties, photostability, etc. [12,13]. It is believed that CDs possess a unique hybrid combination of fluorescence properties of both organic dyes and semiconductor QDs. CDs demonstrate varied emission colors, excellent photostabilities, photoblinking, and photofluctuation properties, which are desired properties for super-resolution imaging. Thus, CDs have been applied as super-resolution imaging probes. 

Good reviews on the fluorescent materials for super-resolution microscopy have appeared in recent years on general fluorescent materials [5,7,8], fluorescent proteins [14], organic dyes [6,15,16], and nanoparticles [17,18]. However, very few works have focused on the application of fluorescent CDs for super-resolution microscopy [19]. Considering the importance of this new kind fluorescent materials—CDs—and their applications for super-resolution microscopy, this review focuses on the development and application of CDs as super-resolution imaging probes, especially in the past five years, with a brief discussion on the theory and principles behind super-resolution imaging techniques. At the same time, the properties of CDs for SRM are discussed. Applications and examples of fluorescent CDs for super-resolution microscopy are presented. Finally, the outlook and conclusions of the field are presented. It is hoped that students and researchers could benefit from this work for developing novel fluorescent probes and propelling applications of SR imaging in extended discoveries. 

## 2. Super-Resolution Techniques

### 2.1. Spatially Patterned Excitation for Super-Resolution Imaging

**Figure 1:**
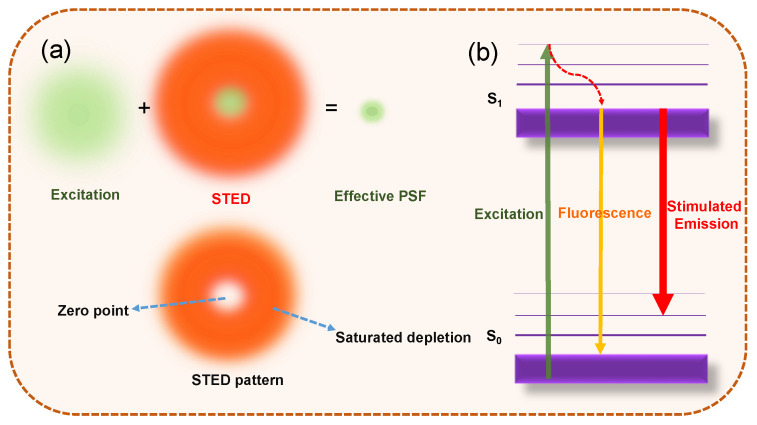
The principle of STED super-resolution microscopy. (**a**) A donut-shaped STED laser is applied with the zero point overlapped with the maximum of the excitation laser focus. With the saturated depletion, the fluorescence out of the zero point is suppressed, decreasing the size of effective point spread function (PSF). (**b**) The Jablonski diagram showing the process of excitation, fluorescence, and stimulated emission. Stimulated emission occurres when the excited-state fluorophore encounters another photon that has a wavelength comparable to the energy difference between the ground and excited state.

The basic principle of STED microscopy is to employ a second laser (called as STED laser or depletion laser), which inhibits fluorescence emission and improves the resolution of the focal plane by depleting the peripheral fluorescence (as shown in Figure 1a) [4,18]. The laser with a donut shape has a pattern with zero intensity at the center of the excitation laser focus and periphery nonzero intensity, typically by inserting a phase mask into the light path to modulate its phase-spatial distribution. Therefore, the fluorescence emission located off the center of the excitation is depleted through stimulated emission (Figure 1a). There is nonlinear dependence of the depleted population on the STED laser intensity when the saturated depletion level is approached. The fluorescence signal could be observed only in a small region around the focal point, reducing the effective width of the point spread function (PSF), avoiding the diffraction barrier and improving the spatial resolution (Figure 1a). The spatial resolution of STED systems is described as

(2) $$\Delta_{\mathrm{eff}}=\frac{\lambda }{2N.A.\;{\left(1+P/P_{\mathrm{sat}}\right)}^{0.5}}=\frac{\Delta }{{\left(1+P/P_{\mathrm{sat}}\right)}^{0.5}}$$

where P represents the power of the STED beam and P_sat_ is the saturated power of the depletion beam when the fluorescence intensity is half of the maximum. The degree of saturation P/P_sat_ could be much higher than unity, and further increasing the ratio is an effective method to improve the resolution. 

Taking advantage of the similar theory, RESOLFT microscopy was designed and developed to achieve super-resolution by taking advantage of saturated depletion. Fluorescent probes are used that could be photoswitched between a fluorescent on state and a dark off state. The gain in spatial resolution is achieved similar to STED, in which a depletion laser is used to darken fluorophores at the periphery of the excitation. For the spatially patterned excitation-based super-resolution approach, it is necessary to have fluorescent probes that are photostable and resistant to photobleaching under the strong STED laser. Otherwise, the probes photobleach easily, which affects the imaging adversely with the collection of too few images. 

### 2.2. Localization-Based Super-Resolution Imaging

**Figure 2:**
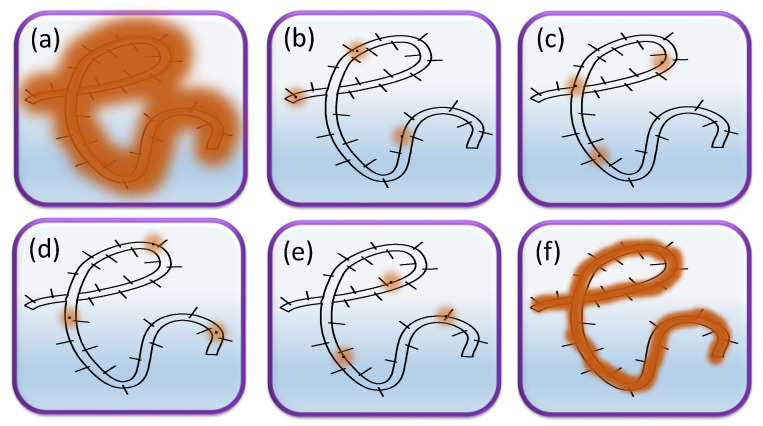
The principle of localization-based super-resolution microscopy. (**a**) Conventional fluorescence image. (**b**–**e**) Single-molecule imaging and localization of fluorescent probe-based fluorescence super-resolution image. (**f**) Reconstruction from positions of many localized probe molecules for super-resolution image.

Molecules within a diffraction-limited region with the fluorescence-on state are imaged at different time points through separating the activation and imaging or tuning blinking properties, such that the activated molecules are optically resolvable from each other, precisely imaged, and localized [7,18]. Massively parallel localization is generally realized through wide-field imaging, and the centroids of many molecules could be mathematically determined in each imaging frame. Super-resolution images are reconstructed via the combination of multiple iterations (Figure 2). Theoretically, PALM/STORM sequentially localizes fluorophores that are randomly switched between fluorescence (“on”) and dark (“off”) by modulating the activation and excitation lasers and then assembles all positions over thousands of cycles for a super-resolution image. PALM/STORM that relies on single-molecule localization almost reaches the ultimate spatial resolution for molecular mapping in a cell, determined by the fluorescent probes. 

For the single-molecule localization-based super-resolution microscopy, the applied fluorescent materials have to satisfy the requirement of photoswitching/photoactivation under the activation laser reversibly or irreversibly or inherent photoblinking, which could help collect multiple images and reconstruct the super-resolution images. To achieve high-quality super-resolution imaging, it is essential that fluorophores have high photon yield for improving localization accuracy and a low on–off duty cycle for maximizing the density of particles [20]. Especially, a low duty cycle could allow the labeling of the probes densely without worrying about the localization accuracy in case multiple signals are present in a diffraction limit spot. 

## 3. Properties of Carbon Dots

### 3.1. Single-Particle Fluorescence Properties

Having a better understanding of single-particle fluorescence on CDs could help identify their photoluminescence mechanisms and obtain information on photoblinking, photoswitching, photostability, and photon counts of single particles [21]. These properties are generally hidden in the ensemble measurements but important and desired for localization-based super-resolution imaging. Photostability properties are especially desired for STED super-resolution imaging probes, which need to withstand the powerful depletion laser. 

Richards et al. investigated CDs fluorescence properties at the single-particle level and discovered single-step photobleaching and transient blinking for CDs [22]. Heterogeneities of CDs were observed with multiple fluorescence intensity levels. The work represents the first time CDs have been studied at the single-particle level and provides invaluable insights into the application of CDs in single-molecule localization-based super-resolution imaging. The same group further investigated the doping and size effect on the single-particle fluorescence intensities [23]. For un-doped CDs, larger particles showed lower intensity, suggesting larger particles favored the non-radiative decay. Doping helped shift the CDs’ emission to longer wavelength even in the near-infrared (NIR) region. However, multiple emissive species were not observed at single particles, and only one emission band was visible [23]. Chizhik et al. collected the single-particle fluorescence spectra of CDs, which resembled those of typical dye molecules. Heterogeneities of CDs were responsible for the broad spectra of CDs solutions at the ensemble level, but varying excitation wavelengths did not shift the emission spectra at the single-particle level [24]. They proposed that CDs behaved as electric dipoles and their emission originated from a charge recombination on surface emission centers involving a strong coupling.

**Figure 3:**
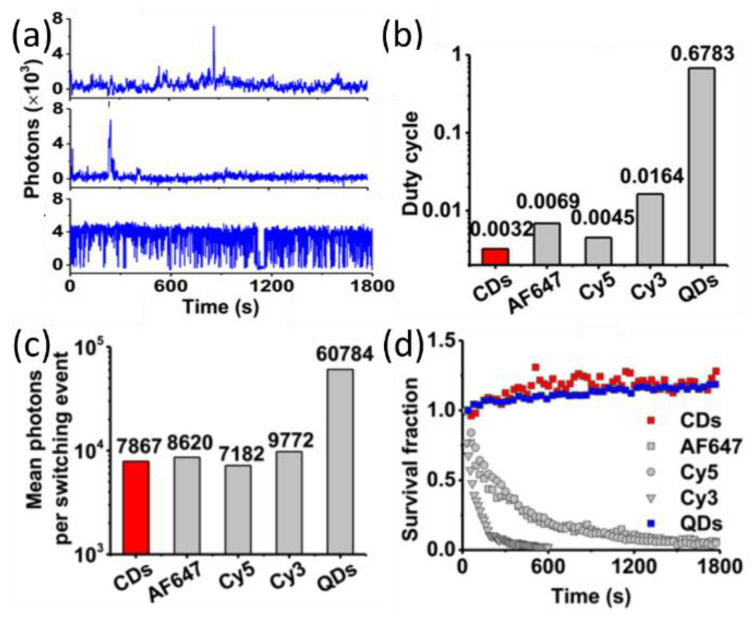
(**a**) Single-molecule/particle intensity trajectories for CDs (top), AF647 (middle), and CdSe/ZnS QDs (bottom). Exposure time was 1 s for CDs and AF647 and 100 ms for CdSe/ZnS QDs. (**b**) On–off duty cycle values, (**c**) mean photons per switching event, and (**d**) number fraction during continuous illumination for each type of fluorophore [25]. Adapted with permission from American Chemical Society, 2017.

The Huang group compared the properties of CDs and other probes at the single-particle/molecule level [25]. The duty cycle is one property that affects labeling density and imaging precisions. Probes with high duty cycle, common in semiconductor QDs, could lead to localization errors, especially leading to multiple fluorescence spots within a diffraction limit area; at the same time, low density of probes results in poor precision imaging. A low duty cycle of probes could allow the labeling of high densities of probes. Photobleaching time and photon counts are indicators to study the fluorescent materials’ photostabilities. The irradiation wavelength and power density affect the photobleaching time, too. Huang et al. developed a type of 4.5 nm CDs with burst-like blinking behavior (in Figure 3a) and demonstrated high-density specific labeling of a microtubule network [25]. They systematically compared CDs with other fluorescent probes and found that the CDs had a low duty cycle (Figure 3b; ~0.003 for CDs versus 0.005–0.016 for organic dyes and rather high values of ~0.7 for QDs), improved brightness (Figure 3c; high photon output of 8000 per switching event), and great photostability (Figure 3d; >30 min, with a few minutes for Cy3). 

Xiao et al. investigated the single-particle intensity traces of polymer CDs and compared the properties with those of QDs and one organic dye (phycoerythrin) [26]. They found that individual CDs showed the brightest intensity and proposed that the brightnesses of single CDs were roughly 10 and 2.6 times those of single QDs and the organic dye. Through analyzing time-resolved single-particle fluorescence intensity, CDs’ photoluminescence stability was studied, and almost no blinking properties were observed, different from control fluorescent probes. Intensity vs. time traces of single particles were generally reported, and the photobleaching time of CDs was affected by dopant and size [23,27]. 

**Figure 4:**
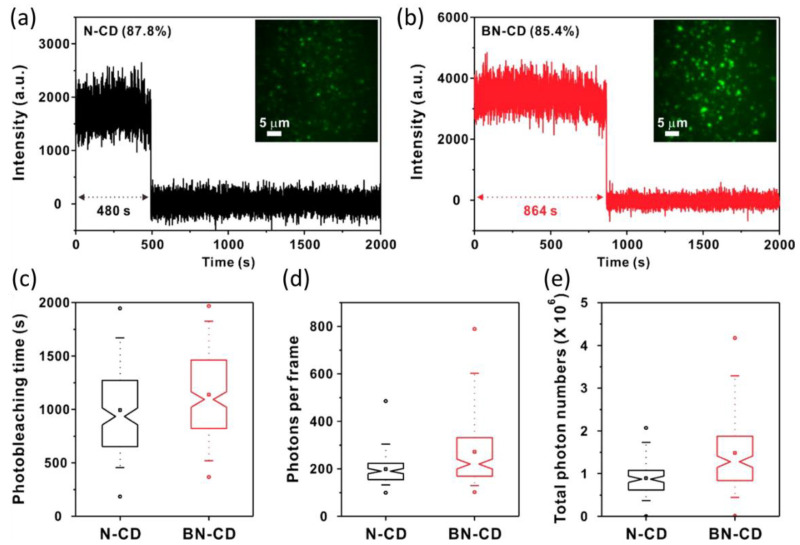
Fluorescence intensity vs. time traces of (**a**) N-CDs and (**b**) BN-CDs. (**c**) Photobleaching time, (**d**) photons per frame, and (**e**) total photon numbers of N-CDs and BN-CDs [27]. Adapted with permission from American Chemical Society, 2016.

Kim and the group investigated properties of nitrogen doped and boron and nitrogen co-doped CDs (N-CD and BN-CD) at the single-particle level [27]. The majority of both CDs demonstrated single-step photobleaching traces (Figure 4a,b). BN-CDs showed modest improvements compared with N-CDs, with longer photobleaching time and increased photon counts (Figure 4c–e). The photon counts of BN-CDs increased by about 60% compared with those of N-CDs. Richards and the group later investigated the size and dopant dependence of the single-particle fluorescence of CDs [22]. Doping of the CDs resulted in changes of the photostability and the fluorescence intermittency at the single-particle level. Milliseconds- to seconds-scale blinking was visualized from red-emitting un-doped CDs, and N-doping obviously reduced blinking. Size-dependent optical properties were observed for both doped and un-doped particles. The photon counts of individual CDs were also affected by dopants, size, and even conjugation groups. Upon conjugation with proteins, the CDs at the single-molecule level showed improved optical properties in photon counts and stabilities [28]. 

Nandi and colleagues studied the origin of CDs fluorescence and photoswitching properties at the single-molecule level [29]. The natural on–off state of carbon dot fluorescence was tuned using an electron acceptor molecule. The photoblinking mechanism of single CDs was investigated through single-particle fluorescence, and charge transfer was proposed to play an important role [30]. 

However, sometimes, the intensity–time traces (proposed from single particles) were not from single particles, which needs to be paid particular attention to. Aggregation of particles may lead to artifacts in photostabilities, photon counts, and photoblinking properties from the proposed single-particle fluorescence studies. In addition to the photophysical properties at the single-particle scale, to serve as appropriate probes for super-resolution imaging, especially of intracellular parts, biological-related properties are required, for example, tuned emission colors, the ability to specifically label the object of interest, biocompatibility, and low cytotoxicity [31]. We summarize and show CDs with these properties in Tables 1 and 2 later. 

### 3.2. Tuned Emission Colors

Most of the early fluorescent CDs demonstrated blue emission colors, either with excitation-independent or excitation-dependent emissions [12,32,33]. For the excitation-dependent emissions, the intensity decreased significantly with extending the excitation wavelength, yielding to the CDs of longer wavelengths with low quantum yields. Recently, researchers developed CDs with multi-emissions, full visible emission colors, or long-wavelength emission (even in infrared emission colors), which are desired in imaging biological structures, as the blue autoluminescence color is prevented [10]. 

The novel CDs with tuned emissions are generally prepared using novel precursors, doping, extending the conjugation, employing fluorogenic reactions, utilizing fluorophore molecules, or efficient separation and purification of CDs mixtures. Red-shifted CDs were prepared with one-pot hydrothermal treatment of precursors of o-phenylenediamine and phenylalanine. The phenylalanine precursor has a benzene ring, and the presence of amino and carboxyl groups helps cross-link, which promotes the formation of carbonaceous graphitic core to form red-shifted fluorescence emission (~584 nm) [34]. p-phenylenediamine with and without the addition of metal catalysts was used to obtain red fluorescent CDs [35]. Further extending the conjugation, diaminonaphthalenes were used as precursors for the preparation of CDs with blue, green, yellow, orange, and red emission colors [36]. Doping of other elements into carbon materials is one option to obtain CDs with novel properties, and bright, dual (blue and green)-emissive CDs were facilely prepared by Lei et al. [37]. 

Inspired by the retrosynthesis and fluorogenic reactions, Xiao et al. obtained rhodamine-based CDs by condensation and carbonization using citric acid and m-aminophenol as precursors [31]. The CDs exhibited excitation/emission peaks at ~575/591 nm, similar to that of rhodamine. Haynes et al. prepared multi-color CDs using solvothermal treatment of citric acid and urea in formamide. Small-molecule fluorophores were proposed to contribute to blue fluorescence, and pyrene analogs were probably responsible for broader emissions from calculated structure as the molecular structures were correlated with optical properties [38]. GQDs were obtained with red to near-infrared emissions, attributed to nanographene molecules and engineering of shape and edge structures [39]. The prepared CDs usually consisted of a mixture of carbon materials; separation and purification were proposed to help obtain materials with improved properties and to understand the photoluminescence mechanisms. CDs in full visible emissions from blue to red were synthesized in one pot and separated via silica gel column chromatography [40]. 

### 3.3. Specific Labeling Properties

CDs can be prepared easily with functional groups such as −OH, −NH_2_, and −COOH on the surface. CDs could have positive or negative charges and help conjugate other molecules or different structures in cells via non-covalent binding such as electrostatic interactions. Pompa et al. observed that CDs were taken up by cells and mostly localized in lysosome compartments, confirmed by colocalization studies [41]. Hynes et al. prepared malic acid carbon dots (MACDs), which displayed two different intracellular distribution patterns under varied excitations [42]. The green-yellow CDs demonstrated filament-like distribution inside live cells and accumulation of CDs in or on mitochondria. The same group later found that different CDs diffused differently inside cells, red CDs localized mainly with lysosomes, blue CDs localized with both lysosomes and mitochondria, and the difference was attributed to hydrodynamic size differences [38]. The mitochondria anchoring ability of CDs was investigated using confocal laser scanning microscopy and suggested good cell permeability of CDs and mitochondria targeting efficiency of CDs in various cells lines [31]. The ability was attributed to electrostatic attraction and hydrogen bond interactions considering the presence of –COOH and –OH on CDs. For example, the CDs effectively accumulated in mitochondria in live cells with selective targeting due to the special physical adhesion and penetration. Amphiphilic carbon dots (Phe-CDs) quickly penetrated into cells and accumulated in the endoplasmic reticulum (ER) mainly through a passive diffusion manner due to their small size and the amphiphilicity property for easy interaction with the amphiphilic phospholipid bilayer membranes [34].

Nucleic acids could also be labeled with CDs. Ni^2+^-phenylenediamine (Ni-pCDs) were found to bind more strongly with RNA than DNA, confirmed by differences of the increase in fluorescence intensities [35]. The targeting specificity was attributed to the structural difference of the two types of nucleic acids, which affected the electrostatic interactions between the positively charged CDs and the negatively charged nucleic acids. The CDs, showing polarity-sensitive and selective RNA-responsive fluorescence properties, also entered the hydrophobic intracellular environment, especially the nucleus, and bound to RNA. Recently, carbonized polymer CDs labeled with nucleic acid resulted in emission enhancement, probably due to reduction of non-radiative transitions from the passivation and fixation effect of nucleic acid molecules to the surface functional groups of CDs [43]. Tuning cationic charges of CDs was also utilized for binding with DNA and RNA in cells [39]. CDs could cross biological barriers from the subcellular to the tissue to the organ level and produce spectrally different fluorescence properties when interacting with DNA or RNA and were thus able to differentiate between DNA and RNA. In this case, p-phenylendiamine (Pda) converted CDs to binding to anionic nucleic acid, and 4-carboxybutyltriphenylphosphonium (PPh^3+^) further increased the cationic charge. dsDNA led to enhanced fluorescence from isolated particles, while ssRNA concentrated CDs and shifted fluorescence into red emissions. 

**Figure 5:**
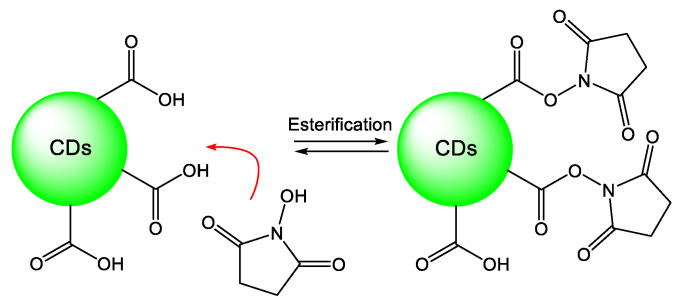
Activation of surface carboxylic acid functional groups of CDs by using NHS to cross-link covalently with the primary amine groups of lysine for specific labeling [44]. Redrawn with permission from Wiley, 2017.

Borrowing the concept of bio-conjugation and covalent bonding, the functional groups on CDs’ surfaces helped specific attachment to different areas/components in cells or were used to further conjugate to some targeting groups or antigens, which enabled specific biomolecular conjugation through biotinylated antibodies. Nandi et al. activated CDs through using N-hydroxysulfosuccinimide (NHS) to react with surface carboxylic acid groups (Figure 5), which cross-linked covalently with the primary amine groups of lysine precisely on proteins [28]. The work provides a way to label any proteins with lysines through CDs with carboxyl functional groups and suggests the antibody labeling specifically for super-resolution imaging in live cells. Later, they used the same coupling chemistry to conjugate between carboxylic acid of CDs and HeLa Cell actin filament specifically [44]. The Huang group also utilized the EDC/NHS strategy for specific labeling of goat antimouse IgG secondary antibody [25], folic acid to recognize folate receptor (FR) mediated endocytosis [45], and Fab fragments of the antibody [46]. 

### 3.4. Biocompatibility and Low Cytotoxicity

Typically, CDs consisted of elements such as carbon, oxygen, and some doping elements (e.g., nitrogen, sulfur, and phosphorus). These were believed to be safe without including toxic heavy metals. In addition, their small size conferred improved biocompatibility, and they were shown to be able to label a variety of subcellular organelles. CDs demonstrated good biocompatibility and low cytotoxicity in both normal cells and cancer cells as the survival rate was high even after incubation with high concentrations of CDs [34,35,39,47]. For example, Phe-CDs demonstrated low cytotoxicity in both normal cells (HEK cell) and cancer cells (HeLa cell), with the classic MTT method at the concentration range of 0.5–20 mg/mL [34]. The CDs’ toxicity experiment also confirmed the viability of cells in the presence of CDs, about 95% viability of prostate cells in high concentrations of CDs (~0.5 mg/mL) and no significant loss in viability of non-malignant cells even at higher concentrations [47]. 

In addition, the low toxicity of CDs was suggested from the high survival rate after co-incubation; a low concentration of the carbonized polymer dots probably promoted the growth of cells and led to insignificant perturbation in the cell cycle [43]. However, when the CDs have short excitation wavelengths, the phototoxicity may need be taken into consideration. As Hynes et al. found upon exposure to blue CDs, enlarged lysosomes were formed in cells, suggesting CDs induced impaired lysosomal fusion–fission balance and higher toxicity. In addition, higher concentrations of CDs may also result in serious death of cells [38]. 

## 4. Super-Resolution Imaging Using CDs

Applications of fluorescent CDs for super-resolution imaging could be generalized into two categories: patterned excitation-based and single-molecule localization-based approaches. 

### 4.1. Patterned Excitation-Based Super-Resolution Techniques

**Figure 6:**
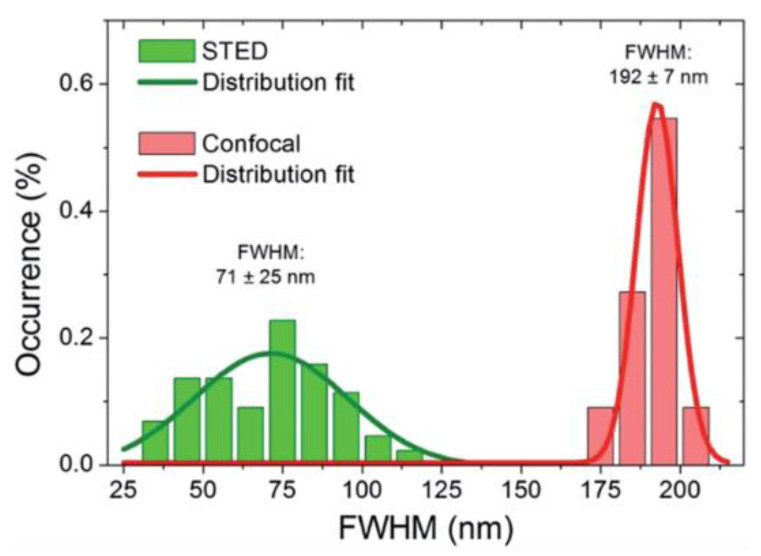
Statistical analysis of FWHM obtained with Gaussian fit of 50 individual emitting spots with STED and confocal microscopy [41]. Reprinted with permission from Royal Society of Chemistry, 2014.

Photostability is one desired property for fluorescent probes for patterned excitation-based super-resolution techniques, such as STED, as the probe needs to withstand the powerful depletion laser. Table 1 summarizes the applications of CDs as STED probes for super-resolution imaging, including the CDs’ properties, imaged bio-system, labeling strategy, and achieved resolutions. Wang and co-workers investigated the CD properties’ potential for STED super-resolution imaging, and the depletion efficiency reached 60% [48]. The CDs showed short emission wavelengths in the violet region, photostability under the excitation power of 100 µW, and weak photobleaching, which demonstrates high potentials to serve as probes for STED microscopy to achieve high spatial resolution. Pompa et al. used diamine-terminated oligomeric (poly-ethylene glycol) functionalized CDs for SR imaging with the STED microscopy [41]. The full width at half-maximum (FWHM) through STED was around 70 nm, compared with ~190 nm for that of confocal microscopy (Figure 6). With the dispersed CDs, a spatial resolution down to 30 nm could be achieved. When CDs were taken up in cells, they were mostly localized in lysosome compartments. Due to the agglomeration of CDs, the spatial resolution of CDs for imaging in both fixed and living cells did not reach as low as that with dispersed CDs but still improved approximately sixfold compared with the diffraction-limited confocal resolution. In living MCF7 cells under physiological conditions, CDs were imaged with a resolution down to 70 nm. 

**Figure 7:**
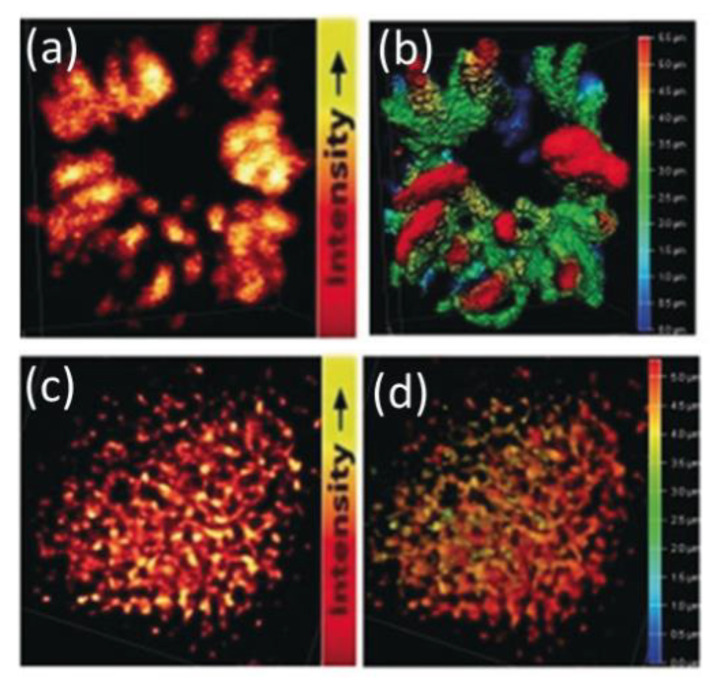
(**a**) STED super-resolution microscopy of chromosomes at prophase (excitation 488 nm, depletion 595 nm, and collection 500–560 nm) and (**b**) a Z-stack STED image of the chromosome’s 3D structure (step 0.12 mm). (**c**) STED microscopy of a nucleolus (excitation 560 nm, depletion 595 nm, and collection 570–650 nm) and (**d**) a Z-stack image of a nucleolus’s 3D structure (step 0.12 mm) [39]. Reprinted with permission from Wiley, 2019.

**Figure 8:**
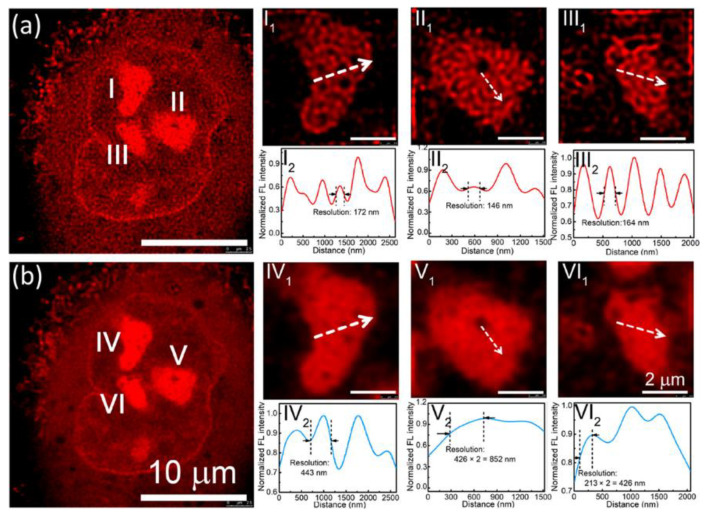
(**a**) STED image and (**b**) confocal image of a representative A549 cell stained by the CDs. (**I_1_**, **II_1_**, and **III_1_**) Enlarged STED images of the nucleoli of the A549 cell in **a** and (**I_2_**, **II_2_**, and **III_2_**) corresponding fluorescence intensity analysis results of the marked lines in **I_1_**, **II_1_**, and **III_1_**. (**IV_1_**, **V_1_**, and **VI_1_**) Enlarged confocal images of the nucleoli of the A549 cell in **b** and (**IV_2_**, **V_2_**, and **VI_2_**) corresponding fluorescence intensity analysis results of the marked lines in **IV_1_**, **V_1_**, and **VI_1_** [35]. Reprinted with permission from American Chemical Society, 2019.

Zhang and others reported on the super-resolution imaging of cell division using STED microscopy with photostable cationic CDs [39]. They demonstrated the CDs’ stability for 10 h even under UV illumination and penetration through various types of barriers in vitro and in vivo. The SR imaging allowed the observation and identification of tangled DNA chains in chromosomes and RNA network in a single nucleolus in HeLa cells. CDs labeled chromosomes at prophase in HeLa cells, and 3D reconstructed models showed tangled DNA chains in chromosomes (Figure 7a,b). The RNA network in a single nucleolus was also clearly visible (Figure 7c,d). The results presented the possibility of using the CD probe to image the ultrastructure in live cells, rather than fix cell imaging by traditional techniques such as transmission electron microscopy (TEM). Wu et al. achieved SR imaging of nucleolus with red-emitting CD-treated cells [35]. The CDs showed not only excellent emission properties (e.g., good photostability and high quantum yield) but also unique intracellular performance such as organelle-specific targeting. They treated the live A549 cells with CDs followed by wash-free STED imaging using 552/660 nm lasers for excitation/stimulated depletion. The enlarged nucleoli showed the full width at half-maximum (FWHM) values of 172, 146, and 164 nm in the STED images (Figure 8), which were about 1/3 of those from confocal images. 

Wu et al. prepared positive-charged CDs with multi-properties. Due to the negative charge on Gram-negative bacteria, CDs were adsorbed on the bacteria’s surface and applied to selective STED imagining of those bacteria [49]. Recently, Qu and others obtained CDs that could stain the nucleolus and tunneling nanotubes (TNTs) in the living cell and were used for STED microscopy [50]. The resolution of the CD particles on the glass surface and the nucleolus in a living cell was around 20 nm. The fluorescence images of TNTs diameter with ~75 nm resolution was achieved in living cell, showing a threefold enhancement compared with that in confocal imaging.

**Figure 9:**
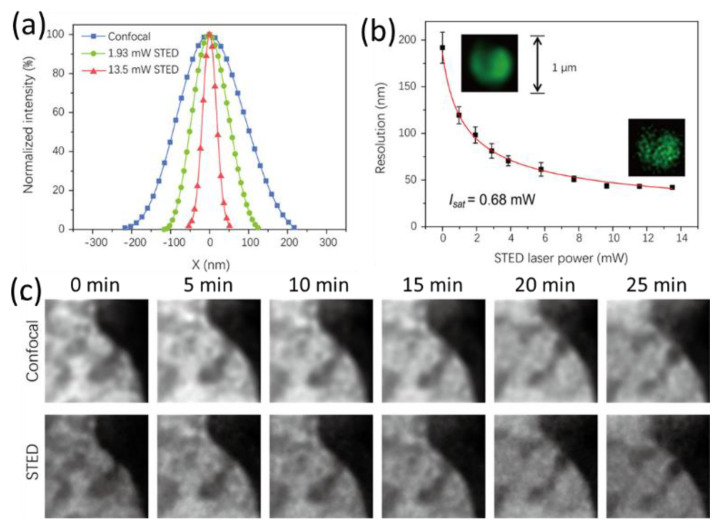
(**a**) Change in measured single-particle resolution for CPDs with increasing depletion laser powers. (**b**) Plot of STED resolution against depletion laser power (calculated I_sat_ at 592 nm: 0.68 mW or 0.23 MW/cm^2^) (inset: the images of particle aggregate of CPDs-3 under confocal and STED imaging modes). (**c**) Time lapse imaging results within 25 min showing the motion of a selected region of a diving cell under confocal and STED modes (STED power as 4.1 mW) [43]. Reproduced with permission from American Chemical Society, 2021.

Later, responsive carbonized polymer dots (CPDs) were used for SR imaging for direct observation of sub-diffraction chromatin structures [43]. The resolution improved as the depletion power increased (Figure 9a,b), consistent with Equation (2). The application of CDs as a long-term probe in the dynamic study and time lapse imaging of a region in dividing cells under STED imaging were achieved, and continuous movement of chromatics was visualized within 25 min (Figure 9c). Very recently, Han et al. reported intracellular distribution of amphiphilic CDs and SR imaging of ER structures [34]. ER tubes and sheets were visualized as a dense network that consisted of a clustering of tubules and the nanoscopic pores within the network with a resolution of ~100 nm. The 3D reconstruction of ER structures from dozens of z-stack photographs with CDs was achieved thanks to photostabilities of CDs. 

**Table 1:** Summarization of applications of CDs as STED probes for super-resolution imaging, including CDs’ properties, imaged bio-system, labeling strategy, and resolution.

Ref.	Emission, nm	Photostability	Excitation Laser, nm	Depletion Laser, nm	Imaged Bio-Systems	Labeling	Resolution, nm
[41] 2014	490	/	405	592	Fixed and live MCF7 cell	Non-covalent interactions	30
[49] 2016	580	/	552	660	Gram-positive bacteria	Electrostatic interactions	~130
[48] 2017	532	1260 s at 100 µW UV excitation	405	532	/	/	/
[39] 2019	510	~10 h under UV illumination	560	595	DNA, RNA Chromosomes and nucleolus	Charge effect	50
[35] 2019,	605	1 h after 552 nm laser irradiation	552	660	Cell nuclei, mice and zebrafish	Electrostatic interactions	146
[50] 2019	513	1000 scan cycles	470	660, 775	Nucleus cytoskeleton	Electrostatic interactions	~20 75
[43] 2021	515	200 frames under 448 nm irradiation	465	592	Nucleic acid, chromatin	Electrostatic interactions	90
[34] 2022	584	100 min continuous UV irradiation	488	660	ER structure and cell division	Non-covalent interactions	100

Notes: “/” indicates not available.

### 4.2. Single-Molecule Localization-Based Super-Resolution Techniques

Fluorescent CDs have been extensively used as probes for localization-based super-resolution microscopy imaging of bio-related targets focused on cellular structures. Table 2 summarizes the application of fluorescent CDs as probes for localization-based super-resolution microscopy, including the CDs’ properties, imaging targets investigated, labeling strategy, and corresponding resolutions. The Nandi group systematically characterized the single-molecule fluorescences of three CDs and compared them with a standard fluorescent probe (Cy3) [33]. CDs dispersed in poly vinyl alcohol (PVA) through spin-coating were used for collecting single-molecule traces and realizing SR imaging. Localization-based super-resolution microscopy with optical reconstruction based on CDs’ blinking was achieved, and a precision of ~35 nm was obtained with dispersed CDs in polymer films. The photon budget of three CDs was in the range of 1500–3000, which was somewhat comparable to the Cy3 dye. A low duty cycle was a desired property for higher resolution through localization-based SR imaging, and the average on–off rate was tuned by the laser power. The blinking properties were attributed to different energy traps and various surface oxidation states. Following that, the Nandi group specifically conjugated one orange emissive CD to a HeLa cell actin filament for STORM and super-resolution radial fluctuation microscopy [44]. For actin specific labeling, the carboxyl functional group of CDs was conjugated with the amine group of phalloidin via 1-ethyl-3-(3-dimethylaminopropyl) carbodiimide (EDC) coupling chemistry. The average localization precision was as low as 25 nm, about one order of magnitude smaller than the diffraction-limited spot. Nandi et al. also imaged nucleolar RNA and actin filament using CDs as super-resolution microscopy [51,52].

**Figure 10:**
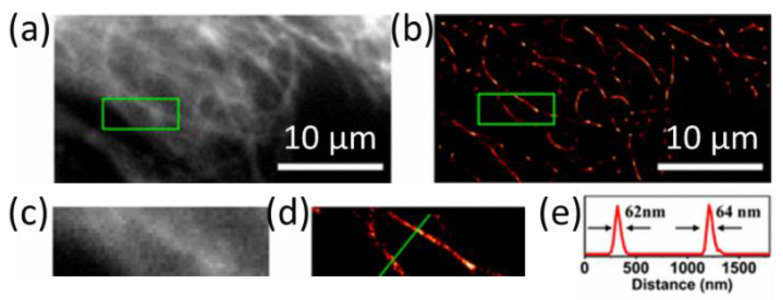
Super-resolution imaging of CD-labeled structures inside a cell and on the plasma membrane. (**a**) The conventional fluorescence and (**b**) the super-resolution image of microtubules labeled with CDs. (**c**,**d**) The enlarged view of small regions in (**a**,**b**). (**e**) The intensity profile of the line region as indicated in (**d**) [25]. Modified with permission from American Chemical Society, 2017.

Huang and others developed a type of 4.5 nm CD with photostability and burst-like blinking behavior and demonstrated the CDs’ use for high-density specific labeling of a microtubule network [25]. The CDs demonstrated a high photon count, a low duty cycle, and good photostability, which make the CDs suitable for localization-based SR imaging and allow the performing of high-density labeling. A resolution of 25 nm was achieved by sequentially locating the positions. The improved resolution by rendering CDs coated on a coverslip, CD-stained tubular peptide self-assemblies, CD-packed clusters with well-defined patterns, and CD-stained microtubules in a cell was demonstrated. CD-stained microtubules in the cell, with improved resolution of the microtubule network, and resolution of ~60 nm were achieved (Figure 10). Therefore, the heterogeneous distribution and aggregation of proteins, hidden under the conventional microscopy, could be visualized. Huang et al. later reported on the graphene oxide nanosheets (GONS)-based CDs serving both as a super-resolution imaging probe and as a drug-bearing nanocarrier [45]. Taking advantage of super-resolution imaging with GONS’s low duty cycle, high photon output, and photostability, they uncovered the distribution of clusters below the diffraction limit, the information on GONS clustering size, distribution density, GONS number in each cluster, and the number fraction of GONSs that participated in clustering at the cell surface and in the cytoplasm and correlated to drug delivery. The graphene CDs thus demonstrated potentials to characterize the receptor-mediated drug delivery through SRM. Recently, the Huang group utilized spontaneous blinking to count single molecules with super-resolution precision of 10 nm [46]. They directly visualized G-protein coupled receptor oligomerization and clustering on the cell membrane and therefore discovered information on the ligand-regulated receptor distribution patterns.

Huang and co-workers also demonstrated SR imaging of HeLa cells using CDs through STORM microscopy [53]. STORM imaging of CD-labeled cells and DNA fibers generated a resolution down to ~60 nm. Although the maximum emission band was centered on the blue range, the 693 nm excitation may have generated the low brightness. Hynes et al. prepared malic acid CDs (MACDs) with blue, cyan, and yellowish-green emissions and further applied the CDs for SR imaging in cells [42]. The MACD’s photoblinking properties, low duty cycle, high photostability, and buffer-independent blinking make them appropriates candidates for improving spatial resolution. The efficient cell uptake of CDs was further utilized for super-resolution imaging in fixed and live trout gill epithelial cells. The resolution improvement could help distinguish closely aligned structures and understand the transportation of CDs in or on mitochondria. Very recently, the Hynes group prepared multicolor CDs and found that different CDs localized differently in cells through SR imaging [38].

**Figure 11:**
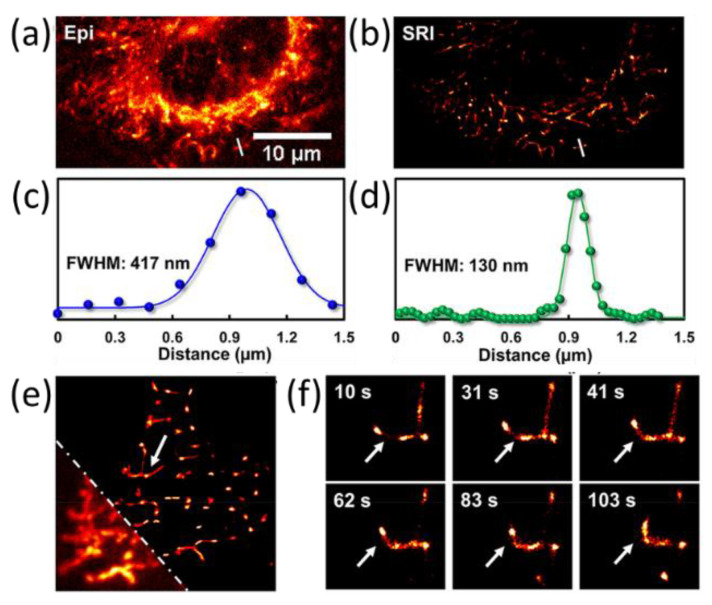
(**a**) Representative conventional (EPI) fluorescence and (**b**) super-resolution imaging (SRI) reconstructed images of mitochondria. (**c**,**d**) Corresponding intensity profiles of the white lines in (**a**,**b**), respectively, and FWHM of 417 nm and 130 nm, respectively. (**e**,**f**) Mitochondrial dynamic tracking with super-resolution imaging [31]. Modified with permission from American Chemical Society, 2019.

Xiao et al. reported on rhodamine-based red fluorescent CDs, with burst-like blinking phenomena and a low duty cycle of ~0.16, which is much smaller than that of QDs [31]. The CDs effectively accumulated in mitochondria in live cells with selective targeting. With the SR imaging, the FWHMs from single mitochondria decreased from 417 nm (diffraction limit) to 130 nm, as shown in Figure 11a–d. Furthermore, the researchers observed the mitochondrial dynamical changes and mitophagy in real time, and fission/fusion of the mitochondrial were clearly visualized beyond the diffraction limit (Figure 11e) as well as tracking in live cells. It is expected to further improve the resolution to tens of nm by tuning density of probes and agglomeration in the cells. The work demonstrated CDs as probes for understanding mitochondria-dependent metabolism at the nanoscale and imaging specific proteins and cellular structures. Very recently, the Xiao group imaged the subtle changes in nucleolar RNA using one carbon dot with RNA anchoring ability and super-resolution microscopy [54].

CDs were also used as SRM probes to show different CDs distributions in surviving bacteria after mild photothermal treatment [47]. In He and others’ work, protein-like CD properties, blinking behavior, and an RNA binding motif endowed the CDs as probes for super-resolution imaging of nucleolar ultrastructure with an enhanced resolution of ~50 nm, and both the STORM and SOFI imaging were studied [55]. Integration of fluorescence resonance electron transfer (FRET) and single-molecule localization microscopy (SMLM) was achieved for SR imaging of microtubules, while CDs in these works served as donors [56].

**Figure 12:**
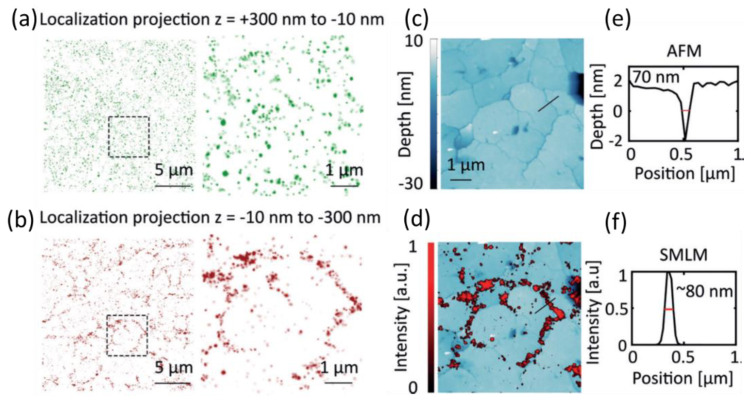
The 3D super-resolution microscopy imaging of nanometer-sized crevices in a glass substrate. (**a**) For the intensity projection along the *z*-axis between 300 and −10 nm above the “etched” structure. (**b**) For the region between −10 and −300 nm, structure of crevices below the glass surface. (**c**) AFM reveals the surface structure in the region of interest in (**a**,**b**). (**d**) Overlay of the SMLM image in (**b**) and AFM image in (**c**) shows strong overlapping features. (**e**,**f**) Line profiles from AFM (**c**) and SMLM (**d**) images [57]. Modified with permission from Wiley, 2019.

Although almost all the CDs for super-resolution microscopy focused on the biological targets, especially the intracellular parts, the abiotic samples/targets were characterized with CDs recently. Liu et al. utilized graphene CDs’ blinking properties to achieve 3D SR imaging of silica nanocracks [57]. The deposition of GQDs (DBOV-Mes (dibenzo[hi,st]ovalene (DBOV) with two mesityl (Mes) groups)) on the coverslips allowed the visualization of crevice features on and below the glass surface. The resulting blinking (from graphene dots) localization of 3D SMLM was classified into two subsets, the distribution of the CDs close to (green) and right below (red) the glass–air interface in the cracks, which were achieved using the bimodal distribution of the localization numbers as a function of the axial distance. The first subset corresponded to the z positions from +300 to −10 nm (Figure 12a), showing a relatively uniform distribution of graphene dots on the surface; Figure 12b presents the distribution of graphene dots with a z position from −10 to −300 nm, indicating the more heterogeneous structure, the etched crevices of the cracks below the glass surface. Highly correlated features at crack locations were obtained from SRM (Figure 12d), consistent with those from atomic force microscopy (AFM) (Figure 12c). Very high spatial resolutions of cracks of ~70 nm and ~80 nm obtained from AFM and SRM were achieved (Figure 12e,f). 

**Table 2:** Summarization of fluorescent CDs as probes for localization-based super-resolution microscopy, including CDs’ properties, applications of bio-system investigated, labeling strategy, and corresponding resolutions.

Ref.	Emission, nm	Photon Counts	Duty Cycle	Excitation, nm	Imaging Targets	Labeling	Resolution, nm
[33] 2016	639	2885	/	532	*E. coli* and HeLa cells	Non-covalent interactions	~35
[25] 2017	590	7876	0.0032	532	Peptide, microtubules, subcellular structures	EDC/NHS reactions	~25
[42] 2018	460	~8000	0.0053	542	Epithelial gill cell	Non-covalent interactions	~36
[45] 2018	480	~3000	~0.003	532	Endocytosis and trafficking drug carriers	EDC/NHS reactions	/
[51] 2018	612	1000 s of	/	560	Nucleolar RNA	Non-covalent interactions	~400
[52] 2018	570	2986	0.0036	532	Actin filament	Non-covalent interactions	~64
[31] 2019	591	~8000	0.16	532	Dynamic fusion from mitochondria in live cells	Electrostatic interactions	130
[44] 2019	612	~6879	/	/	Actin filaments in HeLa cells	EDC coupling	~35
[47] 2019	450	~1200	/	639	Bacteria	/	/
[53] 2019	450	~900	/	647	HeLa cells and DNA fibers	Non-covalent interactions	~60
[57] 2019	701	4960	0.00013	532	Cracking on the glass (non-bio)	Non-covalent interactions	~80
[38] 2021	440/600	/	/	560	Mitochondria and lysosomes	Non-covalent interactions	/
[46] 2021	580	1583	/	488	/	EDC/NHS reactions	/
[55] 2021	580	5171	~0.1	532	Nucleolar ultrastructure	Non-covalent interactions	/
[56] 2021	~600	6100	0.0086	405	Microtubules	Non-covalent interactions	~50
[54] 2022	535	3000	0.06	473	Nucleolar RNA	Non-covalent interactions	~100

Notes: “/” indicates not available.

### 4.3. Image-Correlation Techniques: Super-Resolution Optical Fluctuation Imaging (SOFI)

**Figure 13:**
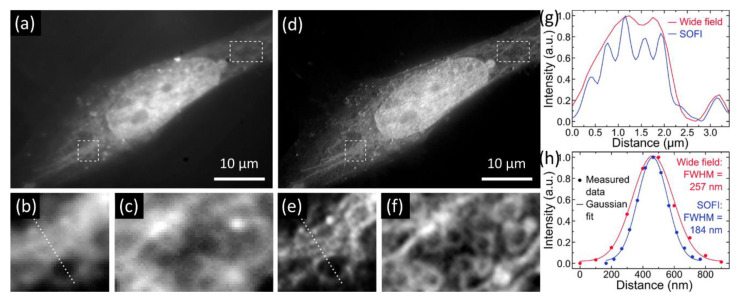
(**a**) Fluorescence wide-field and (**d**) second-order SOFI images of a Saos-2 cell. Two subareas of the cell: (**b**,**c**) wide-field and (**e**,**f**) SOFI images. The dotted lines in (**b**,**e**) indicate cross-sections shown in (**g**). (**g**) Cross-sections through the subimages of the wide-field and SOFI images in (**b**,**e**), respectively. (**h**) Cross-sections through a single bright spot in the wide-field and SOFI image [58]. Modified with permission from American Chemical Society, 2016.

Chizhik et al. demonstrated dual (blue and green) emissive CDs for super-resolution optical fluctuation imaging (SOFI) [58]. As SOFI is based on the temporal fluctuation of a fluorescence signal, they analyzed the blinking statistics of individual CDs. Charge trapping and redistribution on the surface of the CDs triggered their transitions between emissive and dark states. The blue CDs preferentially penetrated the nuclear membrane and labeled the nucleus possibly attributed to the CDs’ charge neutrality. The fibrous, network-like intracellular structures (possibly endosomes and mitochondria) could be localized with the green CDs. There were two sub-regions of the cell where fine subcellular structures were clearly resolved. The SOFI images using the CD probe demonstrated a higher degree of detail compared with the wide-field image as resolution improved ~1.4 times, suggesting the CDs’ potential use for SOFI applications (Figure 13). 

## 5. Conclusions and Outlook

Most of the early CDs showed blue emission colors, which limited their applications in bio-imaging due to overlap with autofluorescence. CDs with multiple or long emissions (in the red or infrared region) demonstrate potentials and have been utilized for the super-resolution imaging of biological structures recently. Red-shifted emissive CDs were obtained with the introduction of precursors to favor the formation of a carbonaceous graphitic core. Several strategies appeared for generating CDs with novel properties such as utilizing novel precursors and dopants, fluorogenic reactions, and separation. CDs with tuned emissions colors especially with long-wavelength emissions were developed for imaging biological samples and sub-cellular structures. 

CDs show high biocompatibility and low cytotoxicity, compared with the famous semiconducting quantum dots. At the same time, they are photostable and demonstrate good survival properties, unlike those of organic dyes or fluorescent proteins. CDs thus could be useful as super-resolution imaging probes (especially for STED) and for the bio-imaging studies (e.g., visualization of cellular events) over long periods of time or in in vivo experiments [41]. Special photoswitching and photoactivation properties, high photon counts, and low duty cycles are desired properties of localization-based SRM. Self-blinking properties without an additional laser to activate or toxic buffers to tune the photoswitching properties are other properties of carbon dots and appropriate for localization-based super-resolution imaging. 

One advantage of using CDs as probes for super-resolution imaging is that they offer extensive potentials for surface functionalization as CDs have functional groups on the surface such as −COOH, −NH_2_, and −OH. Taking advantage of the charges of CDs, the electrostatic interaction between the CD probes and targets is utilized to improve the targeting specificity and was extensively utilized as shown in Table 1 and Table 2. Specific labeling is still a challenge, and it is necessary to further design and develop fluorescent CDs for specific labeling via covalent bond interactions. Recent years have seen CDs with more specific target potentials for subcellular labeling via other mechanisms such as covalent bond interactions and antigen–antibody interactions [25,44,45,59]. The EDC coupling chemistry between the COOH and NH_2_ groups on the CDs and/or the biomolecules of interest is one example. Surface functionalization and conjugation is one approach to enhance the specific targeting and localization. At the same time, the functionalization or conjugation may affect the emission properties of CDs as well [28]. 

Photoluminescence mechanisms of CDs are still under debate. The single-molecule investigation helps understand their PL mechanisms and especially the photoswitching of CDs, which may be masked in the ensemble studies and could extend their applications for super-resolution imaging. Having a better understanding of the mechanisms of the fluorescence blinking of single CDs is one key but has less achieved. Charge-driven fluorescence blinking in CDs may provide a new strategy through the integration of charges for contributing to the localization of super-resolution imaging [30]. The temporal behavior of carbon nanodot blinking follows a power law at both room and cryogenic temperatures. Static quenching via Dexter-type electron transfer between surface groups of a nanoparticle plays a major role in the transition of CDs to off or gray states, whereas the transition back to on states is governed by an electron tunneling from the particle’s core. 

However, potential artifacts may exist in the field as well. Some researchers proposed the single-particle fluorescence of CDs for intensity–time traces of individual particles and obtained blinking and photoswitching properties of single particles. We believe that it is necessary to take a second look at the results as some of the reported single-particle fluorescence results are possibly from the presence of aggregated particles in the targeted area or from impurities. In addition, the aggregation of particles could negatively affect the precision of the resolution in principle.

Further efforts toward the development of fluorescent probes through collaborations among researchers in fields of materials, chemistry, biology, optics, and physics could be helpful not only for improving imaging capabilities but for extending the super-resolution technology to routine use by non-expert researchers for the study of a series of biological issues as well as non-biological ones. CDs and super-resolution imaging are expected to appear in other applications beyond biological studies, such as materials/device characterization, catalysis, and energy fields. Recently, super-resolution imaging of CDs was utilized to investigate the etched, subsurface crevices of cracks, and the results showed consistent overlap with AFM characterizations [57]. It is expected that CDs will open the door to a large number of discoveries in the near future.

## Abbreviations

Super-resolution microscopy	SRM
Stimulated emission depletion	STED
Reversible saturated optically linear fluorescence transition	RESOLFT
Saturated structured illumination microscopy	SSIM
Stochastic optical reconstruction microscopy	STORM
Photoactivated localization microscopy	PALM
Fluorescence photoactivated localization microscopy	FPALM
Super-resolution	SR
Quantum dots	QDs
Carbon Dots	CDs
Graphene quantum dots	GQDs
Point spread function	PSF
Near infrared	NIR
Nitrogen doped	N-CD
Boron and nitrogen co-doped carbon dots	BN-CD
Endoplasmic reticulum	ER
N-hydroxysulfosuccinimide	NHS
Folate receptor	FR
Full width at half-maximum	FWHM
Transmission electron microscopy	TEM
Carbonized polymer dots	CPDs
Poly vinyl alcohol	PVA
1-ethyl-3-(3-dimethylaminopropyl) carbodiimide	EDC
Graphene oxide nanosheets	GONS
Super-resolution imaging	SRI
Fluorescence resonance electron transfer	FRET
Single-molecule localization microscopy	SMLM
Atomic force microscopy	AFM
Super-resolution optical fluctuation imaging	SOFI
p-phenylendiamine	Pda
4-carboxybutyltriphenylphosphonium	PPh^3+^
